# Case Report: Common variable immunodeficiency phenotype and granulomatous–lymphocytic interstitial lung disease with a novel SOCS1 variant

**DOI:** 10.3389/fped.2024.1423858

**Published:** 2024-06-28

**Authors:** María Soledad Caldirola, Espantoso Daiana, Andrea Cecilia Gomez Raccio, Ana Luz García, Agustin Bernacchia, Martín Medín, Maria Isabel Gaillard, Daniela Di Giovanni

**Affiliations:** ^1^ Servicio de Inmunología, Hospital de Niños “Dr. Ricardo Gutiérrez”, Buenos Aires, Argentina; ^2^ Instituto Multidisciplinario de Investigaciones en Patologías Pediátricas (IMIPP—CONICET-GCBA), Buenos Aires, Argentina; ^3^ Servicio de Anatomía Patológica, Hospital de Niños “Dr. Ricardo Gutiérrez”, Buenos Aires, Argentina

**Keywords:** common variable immunodeficiency, granulomatous–lymphocytic interstitial lung disease, SOCS1, GLILD, inborn errors of immunity

## Abstract

Common variable immunodeficiency is a heterogeneous symptomatic group of inborn errors of immunity that mainly affects antibodies production and/or function, predisposing patients to recurrent and severe infections. More than half of them usually develop autoimmunity, lymphoproliferation, enteropathy, and malignancies. Among these conditions, chronic lung disease such as granulomatous–lymphocytic interstitial lung disease is one of the leading causes of death in these patients. Recently, many genes that play a key role in B and T cells’ development, maintenance, and/or cytokines signaling pathways have been implicated in the pathogenesis of the disease. Here, we describe the first Argentinian patient presenting with common variable immunodeficiency and granulomatous–lymphocytic interstitial lung disease, harboring two *in cis* heterozygous variants in the *SOCS1* gene.

## Introduction

Common variable immunodeficiency (CVID) is a heterogeneous symptomatic group of inborn errors of immunity (IEI) that mainly affects antibodies’ production and/or function, predisposing patients to recurrent and severe infections. Classically, CVID is defined in patients over 4 years of age with serum IgG <2 SD for the normal age range and markedly low IgA or IgM isotypes accompanied by a poor response to vaccines and/or absent isohemagglutinins, and in whom other causes of hypogammaglobulinemia have been excluded (1). Although it is frequently diagnosed in young adults, symptoms usually appear in early childhood (2). Moreover, more than half of them usually develop autoimmunity, lymphoproliferation, enteropathy, and malignancies (3, 4). Among these conditions, chronic lung disease such as granulomatous–lymphocytic interstitial lung disease (GLILD) is one of the leading causes of death in these patients (5). Considering the wide range clinical picture of patients, CVID diagnostic criteria has evolved, and in 2019 the European Society for Immunodeficiencies (ESID) added to the published criteria the presence of autoimmunity, polyclonal lymphoproliferation, granulomatous disease, and increased susceptibility to infections (6). Despite being the most prevalent IEI, the genetic cause of CVID is only known in less than 35% of the patients, reinforcing the idea that in most cases there is only a clinical definition of CVID (7).

During recent years, many genes have been implicated in the pathogenesis of CVID, as follows: *ICOS*, *TNFRSF13B*, *TNFRSF13C*, *NFKB1*, *NFKB2*, *CD81*, *CD19*, *IL21R*, *PRKCD*, *CTLA4*, *STAT1*, *STAT3*, *IKZF1*, among others (8–12). These genes play a key role in B and T cells’ development, maintenance, and/or cytokines signaling pathways, confirming the heterogeneous nature of this syndrome (13). If a specific mutation is identified, the patient is reclassified. One of the latest discovered genes associated with immune dysregulation is *SOCS1*. The suppressors of cytokine signaling (SOCS) are a family of essential downregulatory proteins of the Janus kinase and signal transducers and activators of transcription (JAK/STAT) activity. Their encoded protein SOCS1 can directly inhibit JAK kinase activity controlling immune responses to pro-inflammatory cytokines such as interferon gamma (IFN-γ). Moreover, *SOCS1*has the most potent ubiquitin ligase activity of this family that mediates protein's degradation (14, 15).

Recently, SOCS1 haploinsufficiency has been published as a new IEI in a small number of patients with a broad clinical phenotype spectrum, including immunological, rheumatological, and hematological symptoms (16–18). Here, we describe the first Argentinian patient presenting with CVID and GLILD due to a SOCS1 haploinsufficiency.

## Case presentation

A 11-year-old boy, sixth child of non-consanguineous parents, from a rural area in the North of Argentina, with no relevant perinatal history and no vaccines complications, including BCG at birth, was the subject of our case study. His personal history includes vitiligo, an uneventful cat-scratch disease, and asthma as family background. At 8 years of age, he began with abdominal distension and pain. Splenomegaly is noted on physical examination in the absence of other palpable lymph nodes. His first laboratory evaluation revealed leukopenia and thrombocytopenia, mild elevated liver enzymes with normal bilirubin, hypogammaglobulinemia (IgG and IgM <2SD and IgA <1SD for age), normal serum proteins, albumin, and C3 and C4 proteins (Table 1). Stools study was also positive for *Giardia lamblia*. Then, he was referred to our hospital for further examination.

**Table 1:** Laboratory findings.

		Patient (8 years)	Patient (9 years)	Patient (10 years)	Normal range
Initial results	Hemoglobin (g/dl)	12.3	16.3	14.2	11.5–15.5
	Hematocrit (%)	39.0	48.3	45.1	31.0–45.0
	WBC (cells/mm^3^)	**3,000**	6,200	**4,000**	4,500–13,500
	Neutrophils % (mm^3^)	27 (810)	41 (2,542)	40 (1,600)	32–54
	Lymphocytes % (mm^3^)	65 (1,950)	47 (2,914)	40 (1,600)	28–48
	Monocytes (%)	5	11	9	3–6
	Platelets (10^3^/μl)	**69**	181	**148**	150–450
	AST/ALT (UI/L)	**84/125**	28/21	27/15	≤40
	LDH (UI/L)	ND	210	ND	120–300
	Total proteins (g/dl)	6.6	6.8	6.8	6.0–8.0
	Albumin (g/dl)	4.6	4.6	3.9	3.8–5.4
	IgG (mg/dl)	**370**	821^b^	1,170^b^	969–1,485
	IgA (mg/dl)	**30**	**26**	**23**	87–239
	IgM (mg/dl)	**29**	**28**	**21**	68–182
	IgE (UI/ml)	ND	31	ND	≤90
	C3/C4 (mg/dl)	103/12	139/21	104/17	90–150/15–35
	Specific Ig antibodies	**Measles (−)/Mumps (−)** Rubella (+)/Varicella (+)	ND	ND	
	Tetanus toxoid (UI/ml)	0.18	ND	ND	≥0.1
	Pneumococcal (mg/L)	<30	ND	ND	
	Autoantibodies	(−)^a^	(−)^a^	(−)^a^	
	Isohemaglutinins	A (−)/B (1/64)	ND	ND	
Cellular response	CD3% (mm^3^)	73 (2,127)	ND	80 (**1,280**)	55–78
	CD4% (mm^3^)	30 (874)	ND	35 (**560**)	27–53
	CD8% (mm^3^)	37 (1,078)	ND	41 (656)	19–34
	CD19% (mm^3^)	14.6 (425)	ND	7.3 (117)	10–31
	CD56% (mm^3^)	10.0 (291)	ND	10.5 (168)	4–26
	TCR αβ %	94.0	ND	95.1	54–66
	TCR γδ %	6.0	ND	4.7	5–8
	DN TCR αβ CD3^+^ cells %	3.8	ND	3.4	<2.5
	Naive CD4^+^ %	**35.6**	ND	**32.1**	37.8–69.6
	Central memory CD4^+^ %	**53.3**	ND	**51.2**	21.4–40.3
	Effector memory CD4^+^ %	**10.3**	ND	**14.2**	1.5–9.7
	Terminal effector CD4^+^ %	**0.8**	ND	**2.5**	2.6–6.7
	HLA-DR CD4^+^ %	**8.3**	ND	**10.0**	0.6–3.4
	Naive CD8^+^ %	63.9	ND	**38.7**	49.2–82.7
	Central memory CD8^+^ %	**7.0**	ND	**7.7**	11.5–30.7
	Effector memory CD8^+^ %	1.3	ND	4.9	0.7–7.5
	Terminal effector CD8^+^ %	**27.9**	ND	**41.0**	1.5–20.8
	HLA-DR CD8^+^ %	**20.7**	ND	**25.0**	1.4–5.8
	Naive CD19^+^ %	72.0	ND	77.6	
	IgM memory B cells %	24.4	ND	15.7	9.5–17.2
	Switched memory %	**3.3**	ND	**3.4**	5.5–14.9
	Transitional B cells %	**0.1**	ND	8.3	5.1–8.7
	CD21^low^ %	**23.9**	ND	**31.2**	5.6–12.6
	Plasmablast cells %	**0.0**	ND	0.1	0.1–0.2

WBC, white blood cells; ND, not done.

Bold numbers represent abnormal values for age-matched donors. ***T cells* (gated on CD3^+^ CD4^+^ or CD3^+^CD8^+^ cells)**: *Naive T cells* (CD45RA^+^CD27^+^); *Central memory T cells* (CD45RA^−^CD27^+^); *Effector memory T cells* (CD45RA^−^CD27^−^); *Terminal effector T cells* (CD45RA^+^CD27^−^); *Th1 memory* (CD45RA^−^CXCR3^+^ CCR6^−^); *Th2 memory* (CD45RA^−^ CXCR3^−^ CCR6^−^); *Th17 memory* (CD45RA^−^CXCR3^−^ CCR6^−^); ***B cells* (gated on CD19^+^ cells)**: *Naive B cells* (IgM^+^IgD^+^CD27^−^); *IgM memory B cells* (IgM^+^IgD^+^ CD27^+^); *Switched Memory* (IgD^−^IgM^−^CD38^+/−^); *Transitional B cells* (CD38^++^CD24^++^); *CD21^low^ B cells* (CD21^low^CD38^+/−^); *Plasmablasts* (CD38^++^CD27^++^).

^a^ Tested autoantibodies: anti-adrenal, anti-mitochondrial, anti-nuclear antibodies, anti-neutrophil cytoplasmic, anti-parietal cell, anti-*Saccharomyces cerevisiae*, anti-smooth muscle, anti-deaminated gliadin peptide, anti-transglutaminase, anti-glomerular basement membrane, anti-liver-kidney microsome.

^b^ Under IVIG treatment.

**Figure 1:**
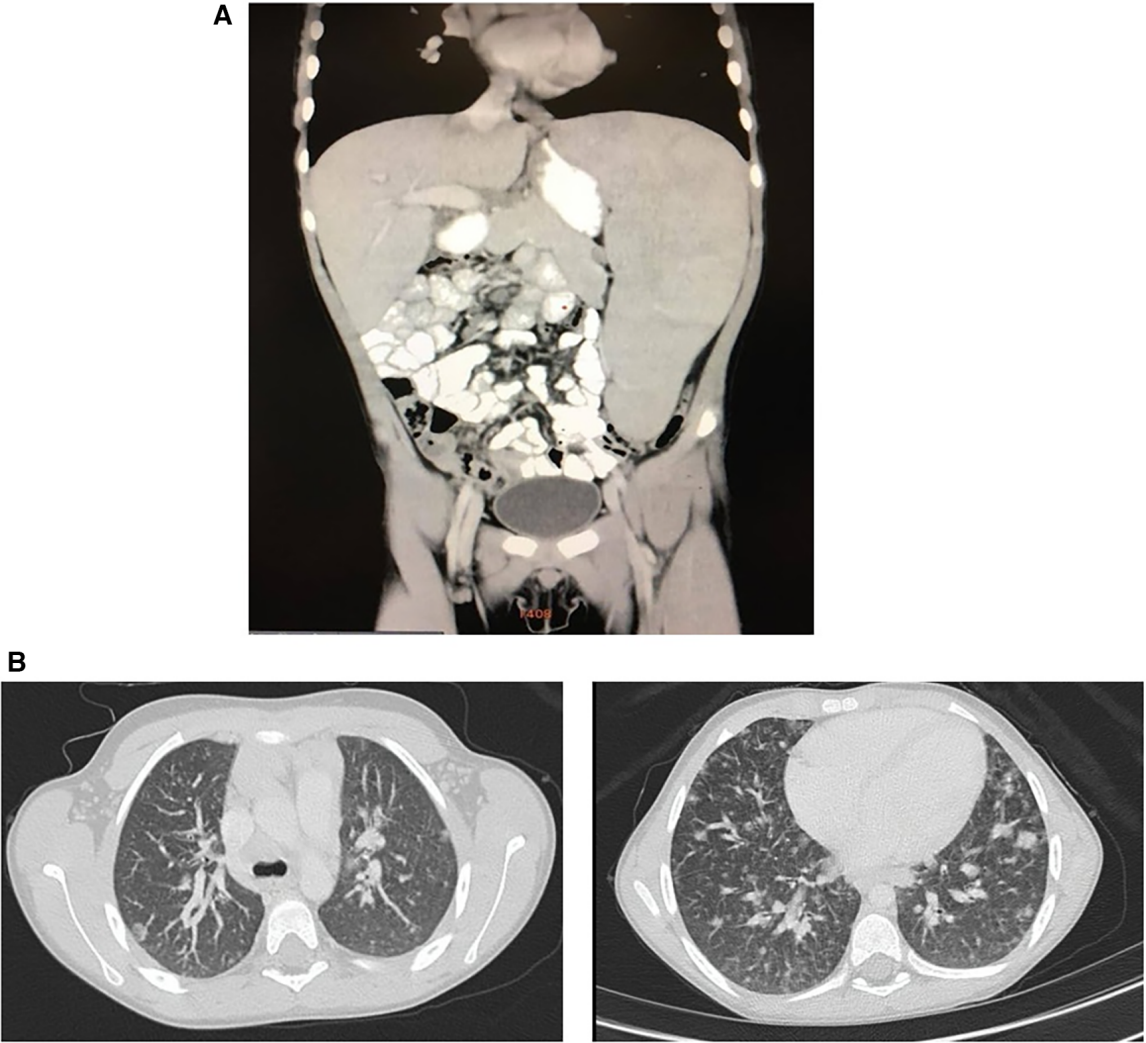
(**A**) Patient abdominal CT scan. Homogeneous craniocaudal splenomegaly measuring 176 mm associated with increased diameter of splenic vascular structures and increased hepatic hilar vasculature. No evidence of free fluid. (**B**) Patient chest CT scan. Bilateral diffusely distributed nodules, including solid, subsolid, and ground-glass opacities, predominantly in the lung bases with the largest measuring 12 mm.

**Figure 2:**
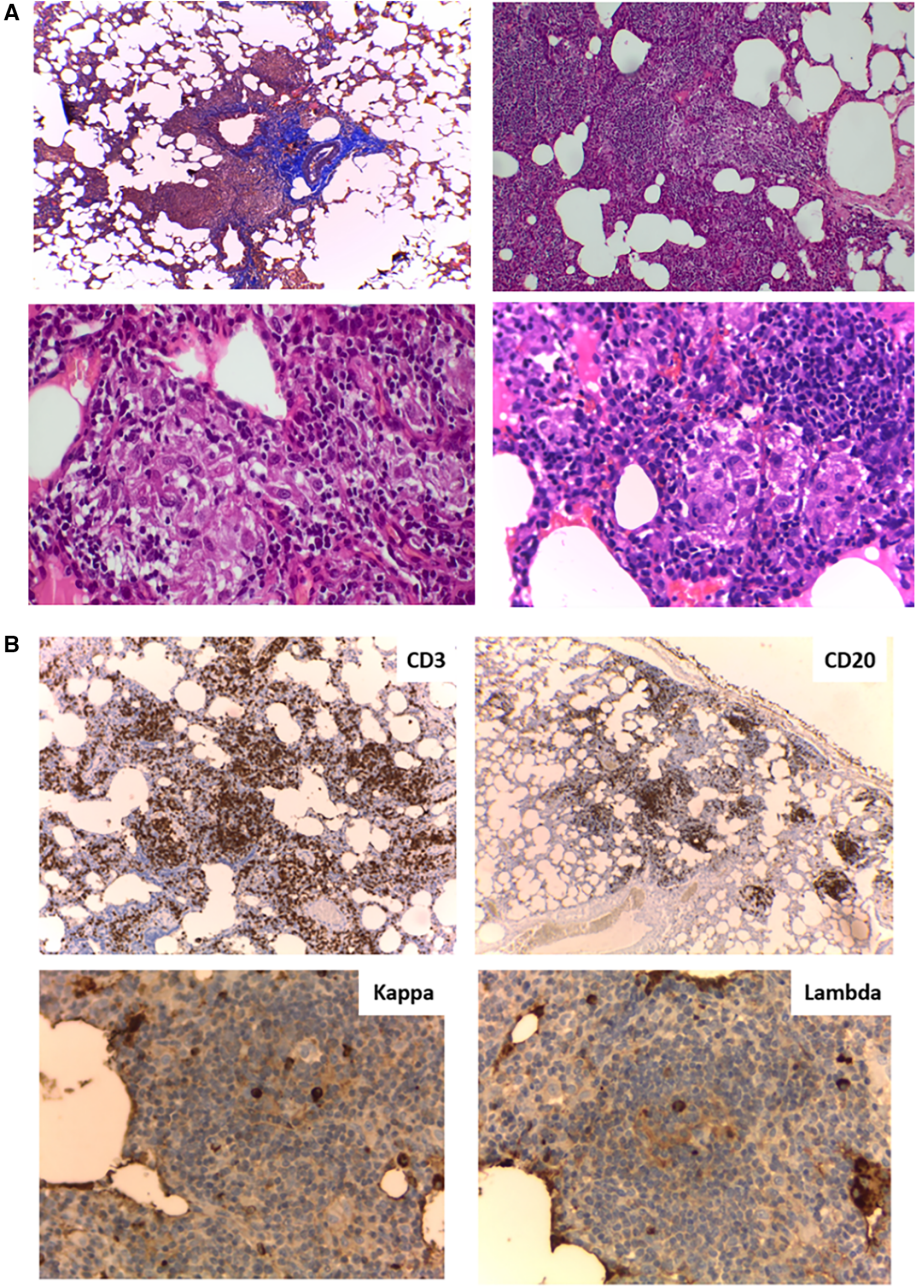
(**A**) Lung biopsy histology. (Upper left) Masson's trichrome stain (4×) peribronchiolar mononuclear inflammatory infiltrate; (Upper right) Hematoxylin and eosin (H&E) stain (10×) Interstitial expansion of lymphocytes and histiocytes; (Lower left and right) H&E stain (40×) Presence of poorly formed non-necrotizing granulomas. (**B**) Lung biopsy immunohistochemistry. (Upper left) (4×) CD3^+^ lymphocytes at the interstitial level; (Upper right) (4×) Aggregates of CD20^+^ lymphocytes; (Lower left and right) (40×) Kappa and lambda chains in similar proportions.

To complete the patient's evaluation, a bone marrow aspirate study was performed, excluding hematological malignancies. CMV, EBV, HIV were discharged by PCR. Abdominal CT scan showed homogeneous splenomegaly (Figure 1A). Regarding pneumological assessment, chest CT scan showed bilateral diffusely distributed nodules, including solid, subsolid, and ground-glass opacities, predominantly in the lung bases (Figure 1B); pulmonary function tests revealed a mixed ventilatory incapacity and low diffusing carbon monoxide (DLCO); and bronchoalveolar lavage showed an abnormal cytological pattern with increased lymphocytes infiltration. Furthermore, lung biopsy was performed showing the presence of a tuberculoid-like necrotizing granuloma, in the superior lobe, and GLILD in the middle lobe (Figure 2A), and immunohistochemistry informed: negative EBERs and CMV, and positive CD3 and CD20 (Figure 2B). Anatomopathological unit ruled out microorganisms with PAS, Ziehl–Neelsen, and Grocott staining. Moreover, cultures for common germs, fungi, and mycobacteria and PCR for *Mycobacterium* complex were done in lungs biopsy and bone marrow with negative results.

Immunological findings confirmed panhypogammaglobulinemia, abnormal polysaccharide response, and impaired response to mumps and measles vaccines, although tetanus toxoid, rubella, and varicella response were within normal range. The results were also normal for T, B, and NK cell subsets count. Low naive T cells with high activation markers expression (HLA-DR) and normal lymphoproliferative response were recorded. The B-cell compartment showed low switched B cells and high frequencies of CD21^low^ (Table 1). Considering these findings, he was diagnosed with CVID and GLILD. He was started on intravenous immunoglobulin (IVIG) replacement and 2 mg/kg/day prednisolone. Considering that the upper lobe lung biopsy evidenced necrotizing granulomatous inflammation, we initiated anti-tuberculosis treatment in the patient based on the high prevalence of tuberculosis in Argentina and the fact that he was about to start immunosuppressive therapy for GLILD treatment, which can increase the risk of reactivation of latent tuberculosis infection, leading to active tuberculosis disease. We added four anti-tuberculous drugs (isoniazid, pyrazinamide, rifampin, and ethambutol) for 2 months followed by a continuation phase with two drugs during 6 months of his treatment.

**Figure 3:**
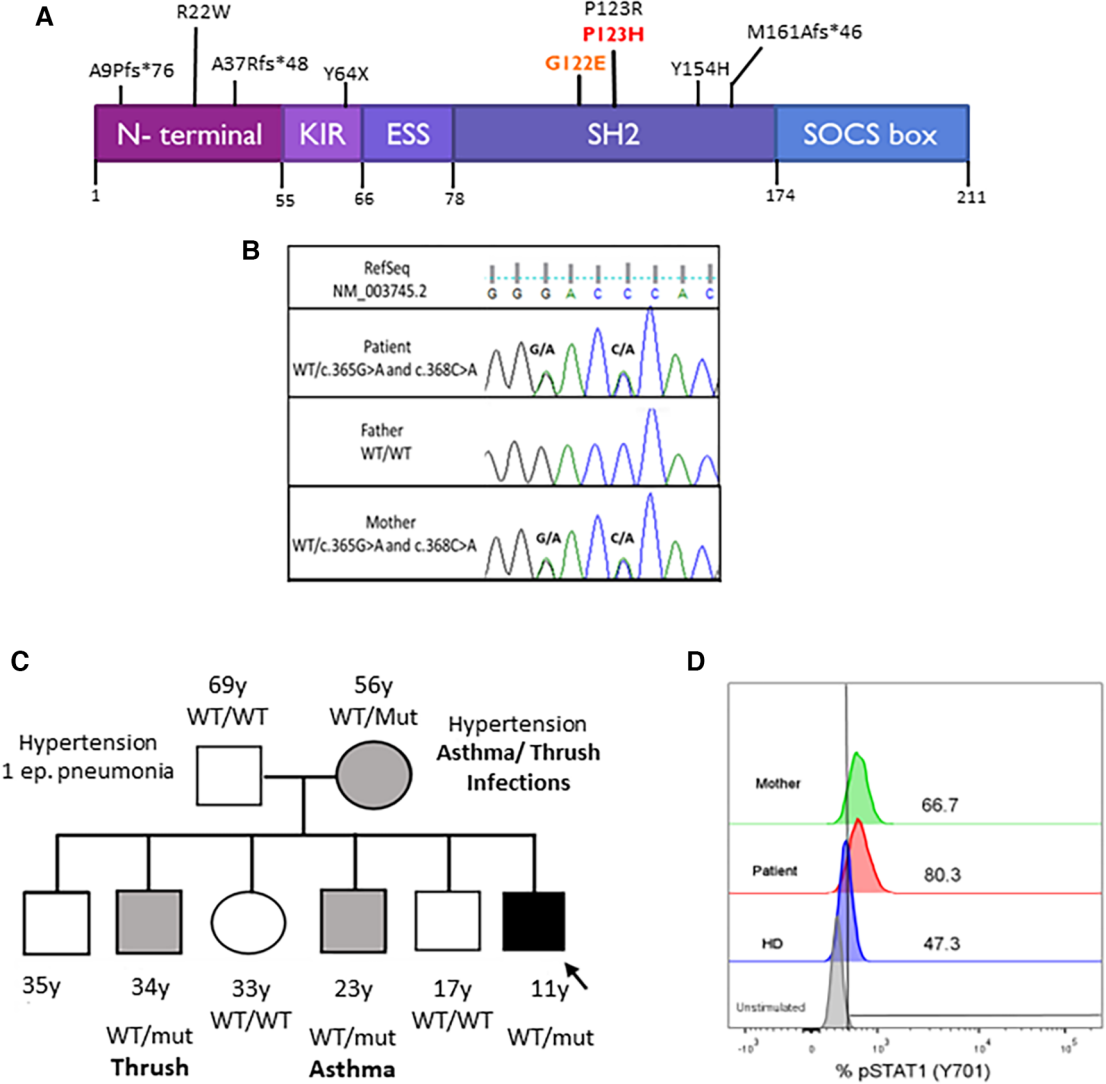
(**A**) SOCS1 protein domains and locations of the variants. The kinase inhibitory region (KIR) functions as a pseudo-substrate that can inhibit the tyrosine kinase activity of JAK proteins. The SRC-homology 2 (SH2) domain binds the activation loop of the JAK proteins’ catalytic domain. The SOCS box recruits the ubiquitin-transferase system and initiates the proteasomal degradation of JAK proteins. Mutations are described in the literature. Our patient's mutations are in red. (**B**) Pedigrees of the family with SOCS1 variants. Squares: males; circles: females; black: affected mutation carriers; gray: unaffected mutation carriers. WT, wild-type *SOCS1* allele. (**C**) Sanger sequencing analysis. Sanger sequencing of the exon 1 of *SOCS1* in the proband and his parents confirmed the maternal origin of: c.365G>A (p.G122E) and c.368C>A (p.P123H) in the same allele of *SOCS1* gene. (**D**) STAT1 phosphorylation assay. Increased phosphorylation of monocytes (CD14^+^) after 15 min with IFN-γ stimulation in the patient and his mother compared with a healthy control (HD).

Whole exome sequencing was performed using the Agilent SureSelect Human Exon Sequence Capture Kit XT v7, Santa Clara, USA. Sequencing libraries were prepared, followed by sequencing on the Illumina platform (Macrogen). Bioinformatic analysis was conducted following the Genome Analysis Toolkit (GATK) Best Practices Workflows, Broad Institute, Cambridge, USA. A total of 106,987 variants were obtained. Variants in genes associated with inborn errors of immunity, clinically overlapping with the proband, were prioritized (Supplementary Appendix S1). Two variants in SOCS1 were identified, NM_003745.2: c.[365G>A; 368C>A] p.[Gly122Glu; Pro123His], in the same allele, with no other clinically significant variants found (Figure 3A). The Gly122Glu variant is not found in gnomAD v4.0, has not been reported in the literature in SOCS1-related conditions, and bioinformatic tools do not predict a deleterious effect (REVEL). The Pro123His variant is also not found in gnomAD v4.0 or the literature. Interestingly, a pathogenic variant in the same codon but with a different amino acid change has been reported before (p.Pro123Arg) (18). Position P123 is highly conserved across vertebrates (philoP100 = 9.74) and deleteriousness of this variant is supported by bioinformatic prediction tools [(REVEL, University of California, Santa Cruz, USA), (CADD, University of Washington, USA)]. Sanger sequencing and familial segregation were performed confirming both variants in the patient, his mother, and two of his brothers (Figure 3B, C). Thereafter, we performed STAT-1 phosphorylation assay in the index case and his mother, which showed enhanced response of the JAK-STAT signaling pathway to IFN-γ stimulus (Figure 3D). The variants p.Gly122Glu and p.Pro123His were classified as variant of uncertain significance and likely pathogenic, respectively, according to the ACMG guidelines.

Following treatment initiation, he experienced improvement in cytopenia, splenomegaly, pulmonary function, and lungs images, and IgG levels. He completed 8 months of anti-tuberculosis treatment, followed by the transition from corticosteroids to mycophenolate mofetil (MMF). This last drug was replaced by rapamycin to address splenomegaly but only for four months as the patient presented severe neutropenia (570 cells/mm^3^) and mild thrombocytopenia (127 × 10^3^/μl). During his treatment, the patient developed a benign SARS-CoV-2 infection. Currently, the patient maintains stable condition with mild, fluctuating thrombocytopenia and moderate splenomegaly under IVIG and MMF. He continues follow-up for euthyroid thyroiditis and slightly elevated calprotectin levels. Notably, the patient reported no history of other remarkable infections besides the one mentioned previously.

## Discussion

Common variable immunodeficiency can present with a variety of non-infectious symptoms that are often misdiagnosed, leading to significant delays in diagnosis and organ sequelae. The clinical course of these manifestations can be more severe than infections in certain instances (19). In the last few years, SOCS1 haploinsufficiency has been described as an autosomal dominant condition with variable expressivity phenotype and incomplete penetrance (16). Since its first description in 2020 in a cohort of 10 patients with early-onset autoimmunity [Evans syndrome, rheumatoid arthritis, systemic lupus erythematosus (SLE), Crohn's disease, psoriasis, type 1 diabetes], only a few studies have been published accounting for less than 20 patients around the globe (16, 18, 20, 21). Moreover, these patients suffered from bacterial infections (otitis media, pneumonia, abscesses), immunodeficiency, multisystem inflammatory syndrome, eczema, and Hodgkin lymphoma, among others. Here we describe the first Argentinian patient presenting with CVID phenotype due to SOCS1 haploinsufficiency.

SOCS1 variants may affect the function of the SOCS1 protein in various ways: some may affect the expression of the SOCS1 protein, while others may affect its function leading to dysregulation of cellular signaling, which may contribute to the development of diseases. Hadjadj et al. reported two patients with heterozygous missense germline SOCS1 mutations in the same amino acidic residue as our patient but with a different amino acidic change. Both individuals experienced immune thrombocytopenic purpura (ITP) from very early onset, with one of them also presenting thyroiditis and polyarthritis (18). Nevertheless, none of them presented with either a CVID phenotype or lymphoproliferation. To date, there are only two reported patients with hypogammaglobulinemia (21) and one patient with CVID with SOCS1 variants in the literature (20). Interestingly, this is a child who developed GLILD and ITP too, although she carries a different variant (p.Met161Alafs*46) that resulted in a SOCS1-truncating protein. Of note, our patient carries two cis variants in the SH2 domain located in contiguous amino acids (p.Gly122Glu and p.Pro123His, respectively). This domain acts as a regulatory checkpoint in intracellular signaling pathways of several cytokines (15). As noted in previous reports, our results also highlight the variable expressivity and incomplete penetrance nature of the disease, as the mother had abnormal laboratory markers compatible with immune dysregulation (Supplementary Table S1) and mild clinical symptoms, whereas both carrier brothers also have mild clinical symptoms. However, we cannot exclude the fact that he may develop other symptoms later in life, and appropriate genetic counseling should be provided. Unfortunately, we were unable to investigate each of them separately, but as mentioned previously, there is a previous report of a pathogenic variant in the same position. In this regard, although the P123H variant is likely to have a major impact on SOCS1 function, we cannot exclude a cis-interacting effect of the other variant, affecting the disease phenotype (22). Due to the limited number of identified cases, research on *SOCS1* variants requires further investigation with a larger patient population to fully elucidate their impact.

Although CVID is traditionally classified as a humoral immunodeficiency, recent years have seen the identification of additional cellular defects contributing to the disease. In this sense, our patient's CD4^+^ and CD8^+^ compartments exhibit a skew toward an activated/memory phenotype, similar to those we and others have described in other diseases with immune dysregulation (23–25). This altered phenotype may arise from the underlying mutation and the enhanced response to interferons as confirmed with STAT1 phosphorylation. In line with this, CD21^low^ B cells express T-bet, a transcription factor that has originally been described as the master regulator of Th1 cell development (26). This population might contribute to the inflammatory symptoms and autoimmune manifestations observed in these patients. Of note, an increased prevalence of these cells has been observed in patients with CVID with autoimmune manifestations, splenomegaly, cytopenia, and other autoimmune diseases such as SLE, like our patient's phenotype (25, 27).

The introduction of Ig replacement therapy has significantly reduced infectious complications in CVID; however, its impact on inflammatory and autoimmune manifestations remains limited, becoming one of the most critical issues in CVID management. Thus, the fine-tuning regulation of cytokine signaling in patients with SOCS1 haploinsufficiency is essential for maintaining a balanced immune system. In this regard, treatment with several immunosuppressive therapies should be included. Steroids, mycophenolate mofetil, hydroxychloroquine, rapamycin, biological agents (rituximab, anti-TNFα), and JAK inhibitors (JAKinhibs) have been reported to control the hyper-inflammatory responses (28). Considering that autoimmune manifestations in patients with SOCS1 haploinsufficiency are frequently observed, compared with infectious presentation, the use of JAKinhibs may be a good option in these patients. They exert their therapeutic effect by targeting a key pathway in cytokine signaling competitively binding to the ATP-binding site of JAK kinases, thereby preventing their activation and subsequent STAT phosphorylation (29). It is well known that one main concern about the use of JAKinhibs is the potential development of severe infections as an adverse effect (30, 31). Nonetheless, as they may cause cytopenia by blocking hematopoiesis via JAK1/JAK2-dependent pathways, the use of these drugs should be under strict consideration and monitoring in each case (32).

In summary, we describe the first case of a patient from Argentina with CVID phenotype and immune dysregulation, due to novel SOCS1 mutation. This case emphasizes the clinical and laboratory heterogeneity in patients with CVID, highlighting the limitations of our current understanding of the underlying mechanisms. Molecular studies are needed to identify dysregulated pathways, paving the way for targeted therapies and improved patient outcomes.

## Data Availability

The original contributions presented in the study are included in the article/**Supplementary Materials**, further inquiries can be directed to the corresponding author.
