# Supplementary material for: Case Report: Common variable immunodeficiency phenotype and granulomatous–lymphocytic interstitial lung disease with a novel SOCS1 variant
Source: Front Pediatr. 2024 Jun 28;12:1423858. doi: 10.3389/fped.2024.1423858 (PMC11239428; doi:10.3389/fped.2024.1423858)
Supplement: Supplementary file 1 [file Datasheet1.pdf]

Supplemental Table 1: Laboratory findings

		**Mother (56 y)**	**Normal Range**
**Initial results**	**Hemoglobin** (g/dl)	14.7	11.5-15.5
	**Hematocrit** (%)	44.4	31.0-45.0
	**WBC** (cells/mm3)	**4300**	4500-13500
	**Neutrophils** % (mm3)	49 (2107)	32-54
	**Lymphocytes** % (mm3)	39 (1677)	28-48
	**Monocytes** (%)	5	3-6
	**Platelets** (10^3/μL)	219	150-450
	**IgG** (mg/dl)	**728**	951-1421
	**IgA** (mg/dl)	**479**	87-239
	**IgM** (mg/dl)	131	68-182
	**IgE** (UI/ml)	ND	≤ 90
	**C3/ C4** (mg/dl)	122 / 24	90-150/15-35
	**Tetanus Toxoid** (UI/ml)	0.86	
	**Autoantibodies**	(+)* 1/160 (Fine speckled)<br>(-)#	
**Cellular response**	**CD3** % (mm^3)	79 (1325)	55-83
	**CD4** % (mm^3)	54 (906)	28-57
	**CD8** % (mm^3)	24 (402)	19-34
	**CD19** % (mm^3)	16.2 (272)	10-31
	**CD56** % (mm^3)	**3.6 (69)**	4-26
	**Naïve CD4$^+$ %**	**24.9**	34.8-70.3
	**Central memory CD4$^+$** %	**59.3**	24.3-42.7
	**Effector memory CD4$^+$** %	**15.5**	2.1-7.4
	**Terminal Effector CD4$^+$** %	**0.3**	2.0-7.2
	**HLA-DR CD4$^+$** %	**22.6**	0.8-2.2
	**Naïve CD8$^+$** %	**11.6**	48.6-87.5
	**Central memory CD8$^+$** %	34.2	9.8-37.6
	**Effector memory CD8$^+$** %	**23.6**	0.2-6.9
	**Terminal Effector CD8$^+$** %	**26.6**	0.8-14.0
	**HLA-DR CD8$^+$** %	**71.1**	1.2-8.1
	**Naïve CD19$^+$** %	46.1	
	**IgM memory B cells** %	**19.7**	10.0-16.8
	**Switched Memory** %	**30.7**	14.0-20.6
	**Transitional B cells** %	0.8	2.3-4.0
	**CD21^{low} %**	**9.7**	< 9.0
	**Plasmablast Cells %**	0.4	0.1-0.2

Abbreviations: *anti-nuclear antibodies, # tested autoantibodies: anti-mitochondrial, anti-parietal cell, anti-smooth muscle, anti-transglutaminase, liver-kidney microsome.

Note: Bold numbers represent abnormal values for age matched donors. *Under IVIG treatment; ND, *not done*; ***T cells* (gated on CD3$^+$ CD4$^+$ or CD3$^+$CD8$^+$ cells):** *Naïve T cells* (CD45RA$^+$CD27$^+$); *Central memory T cells* (CD45RA$^-$CD27$^+$); *Effector memory T cells* (CD45RA$^-$CD27$^-$); *Terminal effector T cells* (CD45RA$^+$CD27$^-$); *Th1 memory* (CD45RA$^-$CXCR3$^+$ CCR6$^-$); *Th2 memory* (CD45RA$^-$CXCR3$^-$ CCR6$^-$); *Th17 memory* (CD45RA$^-$CXCR3$^-$ CCR6$^-$); ***B cells* (gated on CD19$^+$ cells)**: *Naïve B cells* (IgM$^+$IgD$^+$CD27$^-$); *IgM memory B cells* (IgM$^+$IgD$^+$ CD27$^+$); *Switched Memory* (IgD$^-$IgM$^-$CD38$^{+/-}$); *Transitional B cells* (CD38^{++}CD24^{++}); *CD21^{low} B cells* (CD21^{low}CD38$^{+/-}$); *Plasmablasts* (CD38^{++}CD27^{++}).
