# Supplementary material for: Case Report: Common variable immunodeficiency phenotype and granulomatous–lymphocytic interstitial lung disease with a novel SOCS1 variant
Source: Front Pediatr. 2024 Jun 28;12:1423858. doi: 10.3389/fped.2024.1423858 (PMC11239428; doi:10.3389/fped.2024.1423858)
Supplement: Supplementary file 2 [file Datasheet2.pdf]

Supplementary Apendix S1

Whole exome sequencing was performed using the Agilent SureSelect Human Exon Sequence Capture Kit XT v7. Sequencing libraries were prepared, followed by sequencing on the Illumina platform (Macrogen). Bioinformatic analysis was conducted following the Genome Analysis Toolkit (GATK) Best Practices Workflows. A total of 106,987 variants were obtained. Variants in genes associated with inborn errors of immunity, clinically overlapping with the proband, were prioritized:

IEI genes: ACD, ACP5, ACTB, ADA, ADAM17, ADAR, AICDA, AIRE, AK2, ALPI, AP3B1, ARHGEFI, ARPCIB, ATM, ATP6AP1, BACH2, BCLIO, BCLIIB, BLM, BLK, BLNK, BTK, C170RF62, CIQA, CIQB, CIQC, CIS, C2, C3, CARDII, CARD14, CARD9, CASPIO, CASP8, CD19, CD247, CD27, CD3D, CD3E, CD3G, CD40, CD40LG, CD46, CD55, CD59, CD70, CD79A, CD79B, 081, CD8A, CDCA7, CDK9, CEBPE, CECRI, CFB, CFD, CFH, CFI, CFP, CFTR, CHD7, CIITA, CLCN7, CLPB, CDLECII, COPA, ROIA, CR2, CSF2RA, CSF2RB, CSF3R, CTCI, CTLA4, CTPSI, CTSC, CXCR4, CYBA, CYBB, DBRI, DCLREIC, DDX58,GKE, DKCI, DNAJC21, DNASE2, DNMT3B, DOCK2, DOCK8, EFLI, ELANE, EPG5, ERCC6L2, EXTL3, FADD, FAS, FASLG, FCHOI, FERMT3, FOXNI, FOXP3, G6PC3, G6PD, GATA2, GFII, GINSI, HELIS, HYOUI, ICOS, IFIHI, IFNAR2, IFNGRI, IFNGR2, IGLLI, IKBKB, IKZFI, 1110, ILIORA, ILIORB, IL12B, IL12RB1, IL17RA, IL17RC, ILIRN, IL21, IL21R, IL23R, IL2RA, IL2RB, IL2RG, IL36RN, IL6ST, IL7R, IRAK4, IRF2BP2, IRFB, ISG15, ITGB2, ITK, JAGNI, JAKI, JAK3, KRAS, LAMTOR2, LAT, LCK, LIGI, LIG4, LPlN2, LRBA, LYST, MAGTI, MALTI, MAP3K14, MASPI, MEFV, MKLI, MOGS, MREIIA, MSN, MS4A1 (CD20), MTHFDI, MVK, MYD88, MY05A, NBN, NCFI, NCF2, NCF4, NCSTN, NFKBI, NFKB2, NFKBIA, NHEJI, NHP2, NLRC4, NLRPI, NLRP12, NLRP3, NOD2, NOMO, NRAS, NSMCE3, OFDI, ORAII, OTULIN, PARN, PEPD, PGM3, PIGA, PlK3CD, PlK3R1, PLCG2, PMS2, PNP, POLDI, POLE, POLE2, POMP, PRFI, PRG4, PRKCD, PRKDC,PSENEN, PSMB8, PSTPIPI, PTPRC, RAB27A, RAC2, RAGI, RAG2, RASGRPI, RBCKI, RECQL4, RELA, RFX5, RFXANK,RFXAP, RHOH, RIPKI, RLTPR, RMRP, RNASEH2A, RNASEH2B, RNASEH2C, RNF168, RNF31, RNU4ATAC, RORC, RPSA, RTELI, SAMD9, SAMD9L, SAMHDI, SBDS, SERPINGI, SH2DIA, SLC29A3, SLC35C1, SLC37A4, SLC39A7, SLC46A1, SLC7A7, SMARCALI, SMARCD2, SPIIO, SPINK5, SPPL2A, SRP54, SRP72, STATI, STAT2, STAT3, STAT5B, STIMI, STK4, STXII, STXBP2, TAPI, TAP2, TAPBP, TBXI, TCF3, TCN2, TERC, TERT, TFRC, TGFBI, THBD, TINF2, TMC6, TMC8, TMEM173, TNFAlP3, TNFRSF13B, TNFRSF13C, TNFRSFIA, TNFRSF4, TNFRSF9, TNFSF12, TNFRSF7. TRAF31P2, TREXI, TRNTI, TTC37, TTC7A, TYK2, UNC119, UNC13D, UNC93B1, UNG, USBI, USPIB, VAVI, VPS13B, VPS45, WAS, WDRI, WIPFI, WRAP53, XIAP, ZAP70, ZBTB24, ZNF341.

***PIRD genes* :** ABCBI, ACP5, ADA2, ADAM17, ADAR, ADGRE2, AIRE, ANXAII, AP3B1, AP3D1, APOLI, ARID5B, ARPCIB, ATG16L1, ATG5, BANKI, BLK, BTNL2, CIQA, CIQB, CIQC, CIQTNF4, CIR, CIS, C2, C3, C4A, C4B, CALCOC02, CARD14, CARD9, CARMIL2, CASPIO, CASP8, CCDC88B, CCL2, CCL22, CCRI, CCR3, CCR5, CCR9, CD14, CD226, CD27, CD40, CD46, CD55, CD70, CFB, CFH, CFHRI, CFHR2, CFHR3, CFHR4, CFHR5, CFI, CIITA, CLEC16A, CR2, CSK, CTLA4, CTPSI, CXCL13, CXCR5, CYBB, DDX58, DGATI, DNASEI, DNASEIL3, DNMT3A, DOCK8, E2F1 , EGFR, EPCAM, ERAPI, ETSI, FAAP24, FADD, FAS, FASLG, FCGR2A, FCGR2B, FOXP3, FUT2, GUCY2C, HAS2, HNFIA, ICAMI, ICOS, IFIHI, IKZFI, IKZF3, 1110, ILIORA, ILIORB, IL12A, IL12B, IL12RB1, IL12RB2, 1115, IL15RA, IL18R1, ILIRN, IL23A, IL23R, IL2RA, IL36RN, IL7R, IRAKI, IRFI, IRF2BP2, IRF5, IRF7, IRF8, IRGM, ISG15, ITCH, ITGAM, ITK, KIRREL2, KLRC4, KRAS, LACCI, LAT, LCK, LIMK2, LPlN2, LRBA, LYST, MAGTI, MAN2B1, MASP2, MBL2, MC2R, MCM4, MECP2, MEFV, MICA, MICB, MME, MRAP, MSTI, MTHFR, MTMR3, MVK, MYH9, MY05A, MY05B, NCFI, NCF2, NEILI, NEUROG3, NFAT5 , NFKBI,

NFKBIA, NLRC4, NLRP12, NLRP3, NOD2, NPHSI, NROBI, NR4A2, NRAS, OASI, OAS2, OTULIN, PBXI, PDCDI, PDGFRA, PEPD, PLA2R1, PLCG2, PRDMI, PRFI, PRKCD, PRKGI, PSMB8, PSTPIPI, PTEN, PTGS2, PTPN2, PTPN22, PTPRC, PXK, RAB27A, RAG2, RASGRPI, RBCKI, RECQL4, REL, RNASEH2A, RNASEH2B, RNASEH2C, SAAI, SAMHDI, SATBI, SH2DIA, SKIV2L, SLC26A3, SLC7A7, SLC9A3, SPATA5, **SOCS1**, SPINT2, STATI, STAT3, STAT4, STAT5B, STAT6, STK4, STXII, STXBP2, TAGAP, TAPI, TBX21, TCF7, TGFBI, THBD, THBSI, THSD7A, TLR5, TLR7, TLR8, TLR9, TMEM173, TNF, TNFAlP3, TNFRSFIIA, TNFRSF13B, TNFRSFIA, TNFRSF4, TNFSF15, TNFSF4, TNIPI, TREXI, TRIM21, TRNTI, TTC37, TTC7A, TYK2, UBAC2, UBE2L3, UHRFIBPI, UNC13D, WDRI, XIAP, XKR6, ZAP70.

***Additional genes*:** PRRC2A, LILRB5, PSMB9, TNIPI, ARID3A, INPPSD, SH3BP2, BANKI, GAB2, CAMLG, BCL2L11, EBFI, MAGTI, RAG2, SMARCALI, IVNSIABP, PTPN2.

Supplementary S2

		Mother (8 y)	Normal Range
Initial results	**Hemoglobin** (g/dl)	12.3	11.5-15.5
	**Hematocrit** (%)	39.0	31.0-45.0
	**WBC** (cells/mm3)	**3.000**	4.5-13.5
	**Neutrophils** % (mm3)	27 (810)	32-54
	**Lymphocytes** % (mm3)	65 (1950)	28-48
	**Monocytes** (%)	5	3-6
	**Platelets** ($10^3/\mu L$)	**69**	150-450
	**AST/ALT** (UI/L)	**84 / 125**	≤ 40
	**LDH** (UI/L)	ND	120-300
	**Total proteins** (g/dl)	6.6	6.0-8.0
	**Albumin** (g/dl)	4.6	3.8-5.4
	**IgG (mg/dl)**	**370**	969-1485
	**IgA (mg/dl)**	**30**	87-239
	**IgM (mg/dl)**	**29**	68-182
	**IgE (UI/ml)**	ND	≤ 90
	**C3/ C4** (mg/dl)	103 / 12	90-150/15-35
	**Specific Ig antibodies**	**Measles (-)/ Mumps (-)** Rubella (+)/ Varicella (+)	
	**Tetanus Toxoid** (UI/ml)	0.18	>0.1
	**Pneumococcal** (mg/L)	**<30**	
	**Autoantibodies**	(-)#	
	**Isohemaglutinins**	A (-) / B (1/64)	
Cellular response	**CD3** % (mm^3)		55-78
	**CD4** % (mm^3)		27-53
	**CD8** % (mm^3)		19-34
	**CD19** % (mm^3)		10-31
	**CD56** % (mm^3)		4-26
	**TCR αβ** %		54-66
	**TCR γδ** %		5-8
	**DN TCR αβ $CD3^+$ cells** %		< 2.5
	**Naïve $CD4^+$ %**		37.8-69.6
	**Central memory $CD4^+$** %		21.4-40.3
	**Effector memory $CD4^+$** %		1.5-9.7
	**Terminal Effector $CD4^+$** %		2.6-6.7
	**HLA-DR $CD4^+$** %		0.6-3.4
	**Naïve $CD8^+$** %		49.2-82.7
	**Central memory $CD8^+$** %		11.5-30.7
	**Effector memory $CD8^+$** %		0.7-7.5
	**Terminal Effector $CD8^+$** %		1.5-20.8
	**HLA-DR $CD8^+$** %		1.4-5.8
	**Naïve $CD19^+$** %		
	**IgM memory B cells** %		9.5-17.2
	**Switched Memory** %		5.5-14.9
	**Transitional B cells** %		5.1-8.7
	**$CD21^{low}$ %**		5.6-12.6

	**Plasmablast Cells %**	0.1-0.8
